# Dietary inflammatory index and metabolic syndrome in Iranian population (Fasa Persian Cohort Study)

**DOI:** 10.1038/s41598-020-73844-0

**Published:** 2020-10-07

**Authors:** Mohammad Ariya, Hadi Raeisi Shahraki, Mojtaba Farjam, Elham Ehrampoush, Ehsan Bahramali, Reza Homayounfar, Nitin Shivappa, James R. Hebert

**Affiliations:** 1 Noncommunicable Diseases Research Center, Fasa University of Medical Sciences, Fasa, Iran; 2 Department of Epidemiology and Biostatistics, Faculty of Health, Shahrekord University of Medical Sciences, Shahrekord, Iran; 3 National Nutrition and Food Technology Research Institute, Faculty of Nutrition Sciences and Food Technology, Shahid Beheshti University of Medical Sciences, Tehran, Iran; 4 Cancer Prevention and Control Program, Arnold School of Public Health, University of South Carolina, Columbia, USA; 5 Department of Epidemiology and Biostatistics, Arnold School of Public Health, University of South Carolina, Columbia, SC 29208 USA; 6 Connecting Health Innovations LLC, Columbia, SC 29201 USA

**Keywords:** Diseases, Endocrinology, Health care, Medical research, Risk factors

## Abstract

Metabolic syndrome (MetS) is one of the risk factors for all causes of mortality. Inflammation is an important risk factor for MetS. The present cross-sectional study aimed to investigate the relationship between MetS and pro-inflammatory diet by using the food inflammation index (DII). This study consists of 10,017 participants with an age range of 35 to 70 years. The Fasa Cohort Study (FACS) population (Fars Province, Iran) was used to collect data. The DII was estimated according to Shivappa et al. method using a validated 125-item FFQ. To determine the association between MetS components and DII Logistic regression was used (*P* > 0.05). The overall mean of DII was − 0.89 ± 1.74. However, adjusted multinomial logistic regression indicates each unit increase in waist circumference (WC) (OR 0.98, 95% CI 0.96–0.99) and HDL-C (OR 0.99, 95% CI 0.98–0.99) was associated with significantly decreased odds of being in the 4th DII quartile in men and all participations respectively, there is no statistically significant relationship between MetS and DII. Overall, although people in the highest quartile of inflammatory food consumption had more likely to develop MetS, this relationship was not statistically significant among males and females.

## Introduction

Metabolic syndrome (MetS) is one of the risk factors for type 2 diabetes, cardiovascular disease^1^, all causes of mortality^2^, and a major public health problem worldwide^3^. In addition to the main components of MetS, including abdominal obesity, dyslipidemia, hyperglycemia, hypertension, and high-density lipoprotein cholesterol (HDL-C) level decrease^4^, other disorders such as non-alcoholic fatty liver (NAFLD) appear to be a part of the syndrome^5^. Studies have shown that MetS is widely prevalent in Middle Eastern countries, including Iran^5^. According to recent studies, the prevalence of MetS in Iran is estimated to be 36.5%^6^. Also, non-communicable diseases, including MetS, account for a high proportion of deaths in Iran, rising from 57% in 1999 to 76% in 2010^7^.

Although the exact pathology of the MetS is not clear, it seems that genetic background, lifestyle, environmental factors such as dietary habits, physical inactivity, aging, and body mass index (BMI) are important factors in this regard^8^. Additionally, studies have shown that inflammation also is a risk factor for MetS^5,9–11^. Obesity also increases the inflammatory factors that are the underlying causes of MetS^9,12^.

Inflammation is a natural physiological response to cellular damage and a variety of environmental stimuli^13^. In addition to MetS, inflammation is involved in the pathophysiology of many chronic diseases such as obesity, diabetes, cancer, and cardiovascular disease^13,14^. Exposure to chronic stress, on the other hand, leads to low-grade inflammation, which may also play a role in the development of MetS^15^. Although many factors such as age, sex, physical activity, cigarette, and diet play important roles in the development of inflammation, the role of the diet is more evident and well researched^15,16^. Furthermore, studies show that the overall dietary composition for predicting the risk of chronic illness and death is much more important than individual nutrients alone^17^. For instance, some diets such as the Mediterranean diet, which is rich in fruits, olive oil, vitamins, and antioxidants, reduce inflammation, and MetS^18^. In contrast, Western diets, which are rich in a variety of saturated and ω-6 fatty acids and simple carbohydrates, cause the most inflammation among all existing diets^12^.

The Dietary Inflammatory Index (DII) is used to evaluate the anti and pro-inflammatory properties of the diet in different individuals^19^. The index was developed in 2009 and updated again in 2014^19^. So far, more than 200 articles have used this index^20^. It is assumed that the consequences of inflammation can be predicted by this index in virtually any population^5^. This index is designed to assess the inflammatory potential of a diet based on the inflammatory and anti-inflammatory properties of various food components, such as macronutrients, vitamins, minerals, flavonoids, alcohol, and other specific nutritional compounds^21^. It has been reported that the use of this index is very effective in evaluating the level of dietary inflammation^16^. Although various studies in different populations show inconsistent and even non-significant results regarding the DII concerning Mets^22–24^, many studies have reported a significant relationship between MetS or its components and DII to score^5,21,23,25,26^. A recent meta-analysis and systematic review reported that despite the lack of a consistent significant relation between the pro-inflammatory diet and MetS, there is a very strong relationship between the DII and cardiovascular disease (CVD) risk, which is an important sequel of MetS and type II diabetes mellitus^27^.

Based on our knowledge, although a few studies have been done in this regard in Iran, none of them had the same sample size as in our study (10,017 participants). Accordingly, it has been recommended that sample sizes be increased to assess the role of the inflammatory potential of diet associated with MetS and assist policymakers in promoting population health^28^. Therefore, the current cross-sectional study was conducted using data of a cohort study in Fasa (Fars Province, Iran) to examine the relationship between MetS and DII.

## Results

The 10,017 participants had a mean age of 48.64 ± 9.56 years. There were 5499 (54.9%) females and 4518 (45.1%) males enrolled in the current study. Based on BMI, 40.7% of participants were categorized in the normal category, 36.1% were overweight and 17.5% were obese (Table 1). Although the overall proportion of smokers was 27.1%, the proportion of male smokers was significantly higher than females (54.0% vs. 5.1%, *P* < 0.001). Moreover, the overall mean of DII was − 0.89 ± 1.74 which was − 1.33 ± 1.62 and − 0.53 ± 1.76 for males and females respectively (Table 1).

**Table 1:** Basic characteristics of the study participants.

Variable	Male (n = 4518)		Female (n = 5499)		Total (n = 10,017)	
	Number	Percent	Number	Percent	Number	Percent
**Age group (years)**						
< 40	962	21.3	1200	21.8	2162	21.6
40–50	1616	35.8	1894	34.4	3510	35.0
50–60	1216	26.9	1518	27.6	2734	27.3
> 60	724	16.0	887	16.1	1611	16.1
**BMI group (kg/m**^**2**^**)**						
< 18.5	424	9.4	145	2.6	569	5.7
18.5–24.99	2220	49.1	1859	33.8	4079	40.7
25.0–29.99	1457	32.2	2162	39.3	3619	36.1
> 30.0	417	9.2	1333	24.2	1750	17.5
**Smoking status**						
No	2077	46.0	5221	94.9	7298	72.9
Yes	2441	54.0	278	5.1	2719	27.1

	Mean	SD	Mean	SD	Mean	SD
Age (years)	48.63	9.60	48.64	9.53	48.64	9.56
WC (cm)	89.52	11.18	96.13	11.47	93.15	11.81
SBP (mmHg)	110.53	17.68	112.07	19.31	111.37	18.60
DBP (mmHg)	74.34	11.90	74.89	12.17	74.65	12.05
WBC (10^3/µL)	6.55	1.83	6.37	1.69	6.45	1.76
FPG (mg/dl)	90.18	24.22	94.58	33.03	92.59	29.47
BUN (mg/dl)	13.74	3.95	12.29	3.84	12.95	3.95
Cr (mg/dl)	1.06	0.18	0.92	0.18	0.98	0.19
TG (mg/dl)	136.16	91.34	128.28	74.19	131.83	82.46
TC (mg/dl)	178.79	37.96	190.27	39.42	185.09	39.18
HDL-C (mg/dl)	47.24	14.41	54.14	16.39	51.03	15.90
LDL-C (mg/dl)	104.25	31.78	110.44	33.58	107.65	32.92
SGOT (UL/L)	23.85	8.41	21.52	8.43	22.57	8.50
SGPT (UL/L)	26.08	16.52	21.28	12.01	23.44	14.42
ALP (U/L)	211.07	65.56	208.21	75.51	209.50	71.20
GGT (mg/dl)	25.81	22.54	20.42	20.04	22.85	21.37
DII	− 1.33	1.62	− 0.53	1.76	− 0.89	1.74

*BMI* body mass index, *WC* waist circumference, *HDL-C* High-density lipoprotein cholesterol, *SBP* systolic blood pressure, *DBP* diastolic blood pressure, *WBC* white blood cell, *FPG* fasting plasma glucose, *BUN* blood urea nitrogen, *Cr* creatinine, *TG* triglyceride, *TC* total cholesterol, *LDL-C* low-density lipoprotein cholesterol, *SGOT* serum glutamic-oxaloacetic transaminase, *SGPT* serum glutamic-pyruvic transaminase, *ALP* alkaline phosphatase, *GGT* gamma-glutamyl transferase, *DII* Dietary Inflammatory Index.

Results of logistic regression showed that, after adjusting for BMI and age, being in the 3rd or 4th DII quartile was associated with significantly increased odds of MetS (OR 1.20 and 1.37 respectively). Also, among MetS components, DII quartiles were most strongly associated with waist circumference (WC) (Table 2).

**Table 2:** Odds of metabolic syndrome and its component by DII normal quartiles.

DII quartile	Present	Absent	Crude OR (95% CI)	Adjusted OR (95% CI)
**Metabolic syndrome**				
1	522 (23.5)	1982 (25.4)	1.00 (reference)	1.00 (reference)
2	519 (23.4)	1985 (25.5)	0.99 (0.87–1.14)	1.03 (0.89–1.20)
3	549 (24.7)	1956 (25.1)	1.07 (0.93–1.22)	1.20 (1.03–1.39)
4	629 (28.3)	1875 (24.0)	1.27 (1.12–1.45)	1.37 (1.18–1.59)
**Waist component**				
1	1013 (22.7)	1491 (26.9)	1.00 (reference)	1.00 (reference)
2	1096 (24.5)	1408 (25.4)	1.15 (1.02–1.28)	1.43 (1.23–1.66)
3	1129 (25.3)	1376 (24.8)	1.21 (1.08–1.35)	1.95 (1.67–2.27)
4	1227 (27.5)	1277 (23.0)	1.41 (1.26–1.58)	2.70 (2.31–3.16)
**Blood pressure component**				
1	536 (23.1)	1968 (25.6)	1.00 (reference)	1.00 (reference)
2	538 (23.2)	1966 (25.6)	1.01 (0.88–1.15)	1.00 (0.86–1.15)
3	564 (24.3)	1941 (25.2)	1.07 (0.93–1.22)	1.10 (0.95–1.27)
4	685 (29.5)	1819 (23.6)	1.38 (1.21–1.57)	1.28 (1.11–1.47)
**HDL-C component**				
1	1013 (24.0)	1491 (25.7)	1.00 (reference)	1.00 (reference)
2	1022 (24.2)	1482 (25.5)	1.02 (0.91–1.14)	1.04 (0.93–1.17)
3	1071 (25.4)	1434 (24.7)	1.10 (0.98–1.23)	1.16 (1.03–1.30)
4	1110 (26.3)	1394 (24.0)	1.17 (1.05–1.31)	1.29 (1.15–1.44)
**Triglyceride component**				
1	790 (28.0)	1714 (23.8)	1.00 (reference)	1.00 (reference)
2	727 (25.8)	1777 (24.7)	0.89 (0.79–1.00)	0.90 (0.80–1.02)
3	668 (23.7)	1837 (25.5)	0.79 (0.70–0.89)	0.83 (0.73–0.94)
4	637 (22.6)	1867 (25.9)	0.74 (0.65–0.84)	0.77 (0.68–0.88)
**FPG component**				
1	452 (23.6)	2052 (25.3)	1.00 (reference)	1.00 (reference)
2	436 (22.7)	2068 (25.5)	0.96 (0.83–1.11)	0.94 (0.81–1.10)
3	488 (25.4)	2017 (24.9)	1.10 (0.95–1.27)	1.13 (0.97–1.31)
4	543 (28.3)	1961 (24.2)	1.26 (1.09–1.44)	1.16 (1.00–1.34)

Adjusted for BMI and age.

*HDL-C* high-density lipoprotein cholesterol, *FPG* fasting plasma glucose.

As shown in Table 3, although in the unadjusted linear regression model almost all MetS components (except fasting plasma glucose (FPG) in women and men) had a significant relationship with DII, in the adjusted model this relationship only was observed in diastolic blood pressure (DBP), HDL-C and low-density lipoprotein cholesterol (LDL-C) in men and WC, DBP, triglyceride (TG) and HDL-C in women.

**Table 3:** Results of linear regression for the association between DII as a response variable and metabolic syndrome components.

Subgroup	Variables	Model 1 (unadjusted)			Model 2 (adjusted by smoking status, BMI, glucose-lowering medication, and hypertension medications use)		
		Coefficient	Standardized coefficient	*P* value	Coefficient	Standardized coefficient	*P* value
Male	WC	− 0.022	0.002	< 0.001	− 0.009	0.005	0.05
	SBP	0.008	0.002	< 0.001	0.004	0.002	0.10
	DBP	− 0.013	0.003	< 0.001	− 0.008	0.003	0.01
	FPG	− 0.001	0.001	0.43	− 0.002	0.001	0.14
	TG	− 0.001	< 0.001	< 0.001	0.001	< 0.001	0.13
	HDL-C	− 0.006	0.002	< 0.001	− 0.007	0.002	< 0.001
	LDL-C	0.001	0.001	0.04	0.002	0.001	0.03
Female	WC	− 0.17	0.002	< 0.001	− 0.012	0.004	0.01
	SBP	0.012	0.002	< 0.001	0.002	0.002	0.31
	DBP	− 0.014	0.003	< 0.001	− 0.008	0.003	0.02
	FPG	0.001	0.001	0.12	0.001	0.003	0.61
	TG	− 0.001	< 0.001	< 0.001	− 0.001	< 0.001	< 0.001
	HDL-C	− 0.008	0.001	< 0.001	− 0.008	0.001	< 0.001
	LDL-C	0.002	0.001	0.02	0.001	0.001	0.13

*WC* waist circumference, *HDL-C* high-density lipoprotein cholesterol, *SBP* systolic blood pressure, *DBP* diastolic blood pressure, *FPG* fasting plasma glucose, *TG* triglyceride, *LDL-C* low-density lipoprotein cholesterol.

To investigate the effect of each unit increase in metabolic syndrome components on odds of belonging into the 2nd, 3rd, or 4th quartile of DII, multinomial logistic regression was implemented, and results separately were summarized in Table 4. Models adjusted for sex, smoking status, and BMI revealed that each unit increase in WC leads to significantly decrease odds of belonging into the 4th quartile of DII in men (OR 0.98, 95% CI 0.96–0.99). Besides, each unit increase in HDL-C was associated with significantly decreased odds of being in the 4th DII quartile in males and females (OR 0.99, 95% CI 0.98–0.99). It is important to note that these factors marginally were significant. Besides, although the odds ratio (OR) of MetS among all patients in the 4th DII quartile was higher than others, this relationship was not statistically significant among males and females.

**Table 4:** Results of multinomial logistic regression about the effect of each unit increase in metabolic syndrome components on odds of belonging into the 2nd, 3rd or 4th quartile of DII.

Variable	Model 1 (unadjusted)				Model 2 (adjusted by smoking status and BMI)			
	DII normal				DII normal			
	Q1	Q2	Q3	Q4	Q1	Q2	Q3	Q4
**Male**								
Metabolic syndrome	1	1.03 (0.78–1.38)	1.15 (0.84–1.57)	1.24 (0.87–1.79)	1	1.02 (0.76–1.35)	1.16 (0.85–1.59)	1.20 (0.83–1.73)
WC	1	0.99 (0.98–1.00)	0.98 (0.97–0.99)	0.96 (0.95–0.97)	1	0.99 (0.98–1.01)	0.99 (0.98–1.01)	0.98 (0.96–0.99)
SBP	1	1.01 (1.00–1.02)	1.00 (0.99–1.01)	1.02 (1.01–1.03)	1	1.01 (1.00–1.02)	1.00 (1.00–1.01)	1.01 (1.00–1.02)
DBP	1	0.98 (0.97–0.99)	0.99 (0.98–1.00)	0.97 (0.96–0.99)	1	0.99 (0.98–1.00)	0.99 (0.98–1.00)	0.98 (0.97–1.00)
FPG	1	0.99 (0.99–1.00)	1.00 (1.00–1.00)	1.00 (0.99–1.00)	1	0.99 (0.99–1.00)	1.00 (0.99–1.00)	1.00 (0.99–1.00)
TG	1	1.00 (1.00–1.00)	1.00 (1.00–1.00)	1.00 (1.00–1.00)	1	1.00 (1.00–1.00)	1.00 (1.00–1.00)	1.00 (1.00–1.00)
HDL-C	1	0.99 (0.99–1.00)	0.99 (0.99–1.00)	0.99 (0.98–0.99)	1	0.99 (0.99–1.00)	0.99 (0.99–1.00)	0.99 (0.98–0.99)
**Female**								
Metabolic syndrome	1	0.89 (0.71–1.12)	0.97 (0.78–1.21)	1.09 (0.88–1.35)	1	0.88 (0.70–1.11)	0.94 (0.75–1.17)	1.01 (0.81–1.26)
WC	1	0.99 (0.98–1.00)	0.98 (0.98–0.99)	0.97 (0.97–0.98)	1	0.99 (0.98–1.01)	0.99 (0.98–1.01)	0.98 (0.97–1.00)
SBP	1	1.00 (1.00–1.01)	1.01 (1.01–1.02)	1.02 (1.01–1.02)	1	1.00 (0.99–1.01)	1.00 (1.00–1.01)	1.01 (1.00–1.01)
DBP	1	0.99 (0.98–1.01)	0.99 (0.97–1.00)	0.98 (0.97–0.99)	1	0.99 (0.98–1.01)	0.99 (0.98–1.00)	0.99 (0.98–1.00)
FPG	1	1.00 (1.00–1.00)	1.00 (1.00–1.00)	1.00 (1.00–1.00)	1	1.00 (1.00–1.00)	1.00 (1.00–1.00)	1.00 (1.00–1.00)
TG	1	1.00 (1.00–1.00)	1.00 (1.00–1.00)	1.00 (1.00–1.00)	1	1.00 (1.00–1.00)	1.00 (1.00–1.00)	1.00 (1.00–1.00)
HDL-C	1	0.99 (0.99–1.00)	0.99 (0.99–1.00)	0.99 (0.98–0.99)	1	0.99 (0.99–1.00)	0.99 (0.99–1.00)	0.99 (0.98–0.99)

*WC* waist circumference, *SBP* systolic blood pressure, *DBP* diastolic blood pressure, *FPG* fasting plasma glucose, *TG* triglyceride, *HDL-C* high-density lipoprotein cholesterol.

## Discussion and conclusion

In this cross-sectional study, we review the relationship between MetS and DII in rural populations that registered in the Fasa Cohort study (FACS) at Fasa University of Medical Sciences (FUMS). In general, the multinomial logistic regression test shows that people in the highest quartile of inflammatory food consumption, which their dietary habits have the highest level of inflammation risk, are 1.24 and 1.09 times in men and women more likely to develop MetS, which decreases to 1.20 and 1.01 times by adjusting the variables in males and females respectively. These levels are not statistically significant in the present population. Also, the present study showed that men at the highest risk of food inflammation (after adjusting variables) were 0.98 times less likely to be in the group with high levels of WC. We also observed that both males and females the highest risk of DII were 0.99 times less likely to be in the group with high levels of HDL-C. According to Table 4, these results are marginally significant. Other factors related to MetS such as blood pressure, FPG, and TG did not show significant results in any gender.

As noted above, DII is a dietary tool used to evaluate the inflammatory potential of individual diets^19^. This index is a useful tool to predict the level of inflammatory cytokines such as IL6, CRP, and health indices based on the score of dietary inflammation^29^. Studies have shown that higher levels of DII maybe correlate with elevated levels of serum inflammatory factors such as IL-6, CRP, fibrinogen, homocysteine, TNF-α^13,19,23,30^, and white blood cell count (WBC)^31^. In this regard, several studies have reported a significant relationship between diet and inflammatory factor^32^. However, some studies have shown contrary results^33^. Overall, it can be concluded that the nutritional approach can affect the immune response by reducing (or increasing) the rate of these inflammatory indices^34^ and, consequently, lead to the developing or declining incidence of the MetS.

In the present study, an increase in DII score quartiles led to a subsequent rise in odds ratio (ORs) of MetS, but multinomial logistic regression showed that this relationship was not statistically significant among both sexes (Table 4). However, Table 2 shows that after adjusting the variables of age and BMI, this level is significant in the third and fourth quartiles. Other studies show that diets that contain high fats (particularly, saturated and trans fats) and simple carbohydrates or sugar, and a small amount of fiber and omega-3 fatty acids, tend to increase the incidence of non-communication and inflammatory diseases, including MetS^35^. Nevertheless, recent DII studies on the relations between the status of dietary inflammation and various diseases have shown that DII is an appropriate tool for assessing the level of dietary inflammation^16,19^. The results of this study are not consistent with studies that indicate a significant relationship between MetS and DII^5,21,24,36^. In this regard, the results of Spanish cohort studies (n = 6851, 8.3 years)^37^, the cross-sectional studies of Poland-Norway (n = 3862)^22^, Luxembourg (n = 1352)^25^, Lebanon (n = 331)^10^, and the study conducted by American police officers (n = 464)^23^ are consistent with the present study. Finally, a meta-analysis study that examined 17 papers in 2017 reported a lack of any significant correlation between DII and the risk of developing MetS. This study indicates that although the most inflammatory diet, compared to the anti-inflammatory diet, increases the risk of CVD by up to 35%, the risk of developing MetS does not increase^27^. The study also showed that despite the lack of any significant relationship between MetS and DII based on the meta-analysis, there was a significant relationship between DII and other subsets of MetS in some studies^27^.

These differences can be due to the type and design of the study, the target population, race, the number of participants in each study, and how to assess the diet, the type and the number of existing nutrients in the calculation of DII, and the exposure duration of each subset to inflammation. The results of the present study are more reliable than those of previous studies because it was conducted in a cross-sectional design with more target population (n = 10,017). On the other hand, some studies that have reported a significant relationship between these factors emphasize that their results are marginally significant and these results should be interpreted with caution^21^. The sex and age differences of the samples can also be an effective factor in the results of study^24^. According to a study^22^, although there is a significant positive relationship between DII and MetS in women, this relationship is not significant in men.

The relation between MetS and DII may be related to dietary pattern^5^. Various studies have shown the association of dietary patterns with various diseases, including cancer^38^, obesity^39^, diabetes, and MetS. Moreover, it has been reported that the DII score in some diets, including DASH (Dietary Approach to Stop Hypertension), HEI-2010 (Healthy Eating Index-2010), and AHEI (Alternate Healthy Eating Index), is positive^40^. In line with these results, a meta-analysis study showed a significant relationship between MDS (The Mediterranean Diet Score) score and a decrease in blood pressure and TG and an increase in HDL-C^18^. DII score is a specific feature of the existing diet^21^. The correlation between DII and MetS scores relies on the compounds and nutrients that every person consumes during the day, week, or month. In other words, this is the same quality of food consumed by a person and, since this quality varies in different countries, it leads to contradictory results. Another important factor in this context is the number of items used in the FFQ questionnaire or food intake measurement tools, which varies from previous studies and thus leads to different conclusions. Moreover, previous studies have used 55-item^22^, 36-item^21^, and 23-item^24^ 24-h recalls, but we utilized the 125-item FFQ with daily, weekly, monthly, and yearly recalls. In this connection, one meta-analytic study also states that the use of FFQ is much more accurate than 24-h dietary recall^27^. Filling out questionnaires in face-to-face interviews provides more accurate information rather than self-reporting^27^. Therefore, all questionnaires of this study, contrary to some previous studies^37^, have been completed in face-to-face interviews by trained people to minimize the bias of the results.

In the present study, different results were obtained in evaluating the relationship between various parameters of the MetS and the DII, some of which were different and even contradictory in different analyses. For example, WC in the second, third, and fourth quartiles showed a significant positive relationship with the MetS in Table 2. In the adjusted model of a linear regression test, after modulating the variables, this parameter along with HDL-C, DBP, and TG had a significant relationship with DII in women. Finally, multinomial logistic regression showed that by increasing each unit in waist size, the chance of being in the fourth quarter of DII was significantly reduced in men. Neufcourt et al. reported that there was no significant relationship between WC and DII^21^, which was inconsistent with the results of the present study. On the other hand, some studies have shown contradictory results^22,25,26^. The relationship between WC and DII can be interpreted as follows; Since the WC measures abdominal obesity, it can have an important role in the prediction of inflammatory factors^41^. Also, since the WC and abdominal obesity are different in men and women, as well as in different populations, and have different measurement criteria, this may be an important cause of contradiction in the results. In line with this statement, even some studies suggest that there is a direct relationship between DII and abdominal obesity in women; however, this relationship is not statistically significant for men^24^. Hence, it seems that this issue needs further studies.

In the present study, there was a significant relationship between the increase in FPG and DII in Table 2 (in the fourth quartile). However, after adjusting the variables, none of the linear and multinomial regression tests showed a significant relationship among men and women. Unlike some other studies, those from Luxemburg^25^ and France^21^ reported results consistent with the present study. Meanwhile, Nikniaz et al. found a statistically significant relationship between these two parameters in the Iranian population^5^, which is not consistent with the results of the present study. Moreover, the results of the present study are not in line with those reported by Wirth et al. on police officers in the United States^23^. In a Korean study, there was a significant relation between DII and hyperglycemia only in men, while such a relationship was not found in postmenopausal women^24^. It seems that this issue needs further research.

In this cross-sectional study, there was a significant positive correlation between blood pressure and DII in Table 2 in the fourth quartile. Also, in the adjusted linear regression test, DBP was one of the factors that had a significant correlation with DII both in males and females. However, there was no significant relationship between these parameters in the adjusted multinomial logistic regression test. In this regard, the results of some studies are inconsistent with those of the present study^21,26^. It is assumed that inflammation negatively affects the blood vessels and the kidneys and increases blood pressure^42^. Blood pressure, as a multifactorial disease, can be controlled by considering other factors such as environmental factors and stress. The present study and other mentioned studies have not paid attention to this point, which may explain the difference in the results. However, more studies are needed to conclude on this issue.

In our study, there was a significant inverse correlation between DII and HDL-C and TG levels in Table 2 (in the third and fourth quartiles). However, the adjusted multinomial logistic regression showed that each unit increase in HDL-C was associated with significantly decreased odds of being in the 4th DII quartile in both sexes. It is of note that in the adjusted linear regression test, the HDL-C level was among the factors that had a significant correlation with DII both men and women. Besides, TG had a significant relationship with DII in males. However, the findings of some studies were not consistent with those of the present study^21,26^. Meanwhile, a study on 1352 people in Luxembourg reported a significant inverse relationship between the score obtained from DII and the increase in HDL-C levels^33^, which are not consistent with the results of the present study. The French^21^ and the SUVIMAX^37^ studies also yielded results similar to those of the Luxembourg study. Although in this study the dietary patterns and anthropometric factors have been used to assess DII, we should not ignore other factors contributing to the increase of the fat profile, such as inactivity and the type and amount of consumed oil.

The limitations of this study are as follows: first, in this study, the dietary assessment was based on the self-reporting data and the individual’s memory, and the possibility of error in the self-reporting questionnaire was high. To minimize this problem, all questionnaires were filled out by trained people. Second, this study was conducted in a rural area of Fasa. Thus, in other geographic areas in Iran or other countries with different dietary patterns, different results would be obtained. Accordingly, generalizing the results of this study to other populations should be done cautiously. Third, in the present study, only the dietary pattern and DII were emphasized and other parameters such as stress and environmental factors that produce inflammatory factors^43^ were not studied. And finally, in this study, only the correlation between the components of MetS and DII was evaluated and the inflammatory factors were not studied separately. Therefore, this issue should be taken into account in future studies. The other weakness of the present study is that due to the type of study (cross-sectional nature), the causal relationship cannot be derived from the present study.

One of the strengths of the present study is the study design and the sample size. None of the previous studies had a sample size as much as our samples. Also, all previous studies suggested a larger sample size to better understand the relationship between MetS and DII. The present study has all of these features. Another strength of our study is that unlike most previous studies that have used 24-h recalls, in the present study, a 125-item FFQ was used, which is more reliable for dietary pattern assessment. Moreover, all anti-inflammatory foods such as garlic, onions, and spices (e.g., turmeric, saffron, and pepper) were included in this list. Another noteworthy point of this study is that it was conducted by the experts and trained interviewers and all clinical data and tests were carefully recorded. Moreover, the data were recorded electronically for further research on the website ncdrc.fums.ac.ir/collaboration.

To summarize, however, some components of MetS such as DBP, HDL-C, and LDL-C in men and WC, DBP, TG, and HDL-C in women (according to adjusted linear regression) had a significant relationship with DII and also men and all participation at the highest risk of food inflammation (after adjusting variables in multinomial regression) were marginally less likely to be in the group with high levels of WC and HDL-C respectively, our results lack any significant correlation between DII and the risk of developing MetS among males and females (base on adjusted multinomial regression). It seems that to better understand this issue, further analysis on the relationship between MetS and DII in other populations is necessary.

## Materials and methods

The Fasa PERSIAN Cohort study (FACS) population was used to collect data for this study. FACS is a longitudinal study designed to assess the risk factors of non-communicable diseases (NCDs) in Sheshdeh rural district and its 24 surrounding villages (in Fasa County, Iran)^28^. It also is a part of the Prospective Epidemiological Research Studies (PERSIAN) in Iran^44^. A total of 10,017 people aged 35–70 years of Fars, Turk, and Arab ethnicities were recruited into the cohort. People were invited to participate in this study by healthcare experts working in the rural health system. In fact, in Iran, these healthcare experts are called Behvarzes^45^. These experts provide primary health care in small villages and towns. They are residents of the region who are trusted and selected 2 work in the health network of Iran after participating in special courses. This course lasts 2 years and during this time all primary health care and basic physical checkups such as blood pressure control are taught. The experts have an important role in the healthcare system of Iran, and each of them works for a population of 200–2000 individuals at rural health centers. It is noteworthy that before the beginning of the study, all experts participated in the training classes and were fully acquainted with the study protocol, objectives and sampling method. Finally, after counting the households in their district, the experts selected all eligible individuals in each household and invited them to study. The households were selected in a clockwise manner, with the health center as the starting point. In this manner, the health house was selected as the origin and Behvarzes chose every living place for inviting each qualified habitant of the area in a clockwise.

It is important to note that the study protocol was set following the Declaration of Helsinki guidelines and was approved by the Institutional Review Board (IRB) of Fasa University of Medical Sciences (Code: IR.FUMS.REC.1395.177). Written informed consent was obtained from all the participants. Besides, it was confirmed that all the methods were performed following the relevant guidelines and regulations.

### Target area and population

The city of Fasa is located in the eastern part of Fars province, in southwestern Iran. According to the latest population census, the city has a population of about 250,000. The rural area of Sheshdeh with a population of about 41,000 was selected from all surrounding villages of Fasa County. A population with an age range of 35 to 70 years was chosen as the target population for participation in this study. The main reason for selecting this population is that this demographic group, on one hand, is old enough to be exposed to health problems and risk factors for non-communicable diseases and, on the other hand, it is young enough that the final complications of CVD and other NCDs, including MetS, have not yet occurred.

### Management office, sampling, and laboratory

In the present study, an office was provided in Sheshdeh and at the main health center of the region for sampling and interviewing the participants. Also, computers and the equipment needed for the interview and sampling were provided. The study laboratory was located at the Center for Research on NCDs in Fasa University of Medical Sciences (FUMS).

### Human resources

A team of supervisors, two physicians, interviewers, nurses, blood sampling technicians, a driver, and a guard were working at the office daily. The duty of the supervisor was monitoring and performing sampling based on the protocol. All team members were selected from among volunteers and recruited after passing special courses. The interviewers were trained to complete the questionnaires of the participants in the form of a standard digital questionnaire and screening tool, based on the participants’ answers. To increase efficiency, according to the questionnaire type, the interviewers were divided into two groups: public information and nutrition information groups.

### The study procedure

Initially, the registration began with the national ID registration (IDID), and then a proprietary code (PCID) was given to each participant. To identify the individuals, they were asked to use this code in questionnaires and forms until the end of the research. Before starting the research, the written informed consent form was signed by all participants. In the next step, height, weight, and body composition were measured using BIA [(Bioelectrical impedance analysis (Tanita BC-418, Japan)] for all participants and BMI was calculated by dividing the weight by height squared. Also, the WC of all participants was measured. Then, blood pressure was measured by a trained nurse after a 15-min rest in a sitting position (sphygmomanometer, mercury, ALPK1, Japan). For accuracy, this evaluation was repeated after 15 min and the mean of both measurements was reported as the blood pressure of each subject.

Next, after a 10–14-h overnight fast, a 20 ml blood sample was obtained from each participant, and these were stored in encoded tubes after sampling. Afterward, tubes were referred to a laboratory of NCDs to perform relevant tests [WBC, FPG, Blood urea nitrogen (BUN), Creatinine (Cr), TG, total cholesterol (TC), serum glutamic-oxaloacetic transaminase (SGOT), Serum glutamic-pyruvic transaminase (SGPT), alkaline phosphatase (ALP), HDL-C, gamma-glutamyl transferase (GGT)]. All tests (including FPG) were done after at least 10 h of fasting and were measured by Pars Azmoon kits (Pars Azmoon Co., Tehran, Iran).

FPG was measured by using the glucose oxidase method, GGT by enzymatic method base on SZASZ's approach, and SGOT, SGPT, and ALP by the kinetic method. BUN concentration was determined according to the manufacturer’s instructions and Serum Cr was measured by Jaffe’s method. HDL-C, TG, and total cholesterol levels were also determined by the enzymatic method and were recorded in mg/dl. Besides, the Friedewald formula (FF) was used to assess LDL-C^46^.

According to the definition of the National Cholesterol Education Program’s Adult Treatment Panel (ATP) III, if the person has 3 following conditions were considered as the MetS: TG ≥ 150 mg/dl, HDL-C < 40 mg/dl for male, and < 50 mg/dl for females, FPG > 100, systolic blood pressure (SBP) > 130 mmHg and/or DBP > 85 mmHg, and WC > 88 cm for women or WC > 102 cm for men^47^.

### Interview to fill out the questionnaire

As previously mentioned, this stage was divided into two parts. In the general questionnaire, the demographic information of the participants was recorded. This part includes personal information, socioeconomic status, occupational status, residence, lifestyle, smoking, anthropometric measurements.

A 125-item Food Frequency Questionnaire (FFQ) based on the Willett-type questionnaire with necessary changes made based on the Iranian food regime was used to assess the dietary habits and type of food consumed by participants in the last year. People were asked to report the frequency and amount of each food on the list (Iranian foods) on a daily, weekly, monthly, and annual bases over the past year. The standard size of each food is based on the USDA (United States Department of Agriculture) values More details are available at ncdrc.fums.ac.ir/collaboration)^28^.

### How to fill out and scoring the DII questionnaire

Dietary Inflammatory Index (DII) scores were calculated according to Shivappa et al., the complete description of which is available elsewhere^19^. The DII was based on determining which food parameters were documents in the peer-reviewed literature to have affected at least one of 6 inflammatory factors (a significant increase in IL-1β, IL-6, TNF-α, and CRP or a significant decrease in IL-4 or IL-10)^19^. A total of 45 food parameters were identified in a literature search of 1943 articles. A score of + 1 was assigned to a positive association with a pro-inflammatory biomarker or a negative association with an anti-inflammatory biomarker. A score of -1 was assigned to a negative association with a pro-inflammatory biomarker or a positive association with an anti-inflammatory biomarker. A zero score was assigned to all null results^19^. A Z-score was calculated and transformed into a centered percentile score. The DII score was multiplied by the overall score of the food parameter to obtain a specific score of the food parameter. Finally, the E-DII score of each person was calculated by summing all the specific scores of the food parameters. As for the individual scores, positive and negative overall DII/E-DII scores represent the pro-inflammatory and anti-inflammatory dietary patterns, respectively.

In the present study, the average daily intake of 125 food parameters was measured using a validated Quantitative Food Frequency Questionnaire (FFQ). The food parameters calculated in the present study are as follow.

Alcohol (g), Vitamin B12 (ug), Vitamin B6 (mg), Beta Carotene (ug), Caffeine (g), Carbohydrate (g), Cholesterol (mg), Energy (kcal), Eugenol (mg), Total Fat (g), Fiber (g), Folic Acid (ug), Garlic (g), Ginger (g), Iron (mg), Magnesium (mg), MUFA (g), Niacin (mg), Omega 3 (g), Omega 6 (g), Onion (g), Protein (g), PUFA (g), Riboflavin (mg), Saffron (g), Saturated Fat (g), Selenium (ug), Thiamin (mg), Trans Fat (g), Turmeric (mg), Vitamin A (RE), Vitamin C (mg), Vitamin D (ug), Vitamin E (mg), Zinc (mg), Green tea/Black tea (g), Flavan-3-ol (mg), Flavones (mg), Flavonols (mg), Flavonones (mg), Anthocyanidins (mg), Isoflavones (mg), Pepper (g), Thyme/Oregano (mg), Rosemary (mg).

### Statistical methods

Descriptive statistics for quantitative variables were reported as number (%) and for quantitative variables as mean ± SD. To assess the association between MetS components and DII as a response variable, linear regression was used and adjusted odds of MetS and its component by DII normal quartiles were investigated via logistic regression models. Finally, the separate effect of each MetS component on DII quartiles was investigated via multinomial logistic regression. All the statistical analysis performed in SPSS 19.0 software and *P* < 0.05 was considered as statistically significant.

### Ethics approval and consent to participate

The study protocol was following the Helsinki Declaration and was confirmed by the Ethics Committee of Fasa University of Medical Sciences (Approval Code: IR.FUMS.REC.1395.177). The participants were informed about the research objectives and the written informed consent was obtained from the subjects before starting the survey.

### Consent for publication

Not applicable.

## Data Availability

The datasets used and/or analyzed during the current study are available from the corresponding author on reasonable request to the corresponding author.
